# Distinct Roles of IL-1β and IL-18 in NLRC4-Induced Autoinflammation

**DOI:** 10.3389/fimmu.2020.591713

**Published:** 2020-10-14

**Authors:** Yuki Sasaki, Kunihiro Otsuka, Hideki Arimochi, Shin-Ichi Tsukumo, Koji Yasutomo

**Affiliations:** ^1^ Department of Immunology and Parasitology, Graduate School of Medicine, Tokushima University, Tokushima, Japan; ^2^ Department of Interdisciplinary Researches for Medicine and Photonics, Institute of Post-LED Photonics, Tokushima University, Tokushima, Japan; ^3^ The Research Cluster Program on Immunological Diseases, Tokushima University, Tokushima, Japan

**Keywords:** autoinflammation, NLRC4, interleukin-1β, interleukin-18, bone

## Abstract

The NLRC4 inflammasome assembles in response to detection of bacterial invasion, and NLRC4 activation leads to the production of IL-1β and IL-18 together with pyroptosis-mediated cell death. Missense activating mutations in *NLRC4* cause autoinflammatory disorders whose symptoms are distinctly dependent on the site of mutation and other aspects of the genetic background. To determine the involvement of IL-1β and IL-18 in the inflammation induced by *NLRC4* mutation, we depleted IL-1β, IL-18, or both cytokines in Nlrc4-transgenic mice in which mutant *Nlrc4* is expressed under the MHC class II promoter (Nlrc4-H443P-Tg mice). The deletion of the *Il1b* or *Il18* gene in Nlrc4-H443P-Tg mice reduced the neutrophil numbers in the spleen, and mice with deletion of both genes had an equivalent number of neutrophils compared to wild-type mice. Deletion of *Il1b* ameliorated but did not eliminate bone marrow hyperplasia, while mice deficient in *Il18* showed no bone marrow hyperplasia. In contrast, tail bone deformity remained in the presence of *Il18* deficiency, but *Il1b* deficiency completely abolished bone deformity. The decreased bone density in Nlrc4-H443P-Tg mice was counteracted by *Il1b* but not *Il18* deficiency. Our results demonstrate the distinct effects of IL-1β and IL-18 on NLRC4-induced inflammation among tissues, which suggests that blockers for each cytokine should be utilized depending on the site of inflammation.

## Introduction

Inflammasomes are composed of an assembler protein such as a nucleotide-binding domain-containing proteins, a leucine-rich family protein, ASC and caspase-1, and inflammasome stimulation leads to the maturation and secretion of the IL-1β and IL-18 cytokines (1–3). Inflammasome activation is induced by pathogen-associated molecular patterns as well as damage-associated molecular patterns. The activation of the inflammasome is accompanied by pyroptosis-mediated cell death, which causes burst secretion of IL-1β and IL-18 (1, 4).

Hyperactivation of inflammasomes causes several types of diseases called autoinflammatory disorders (5–8). *NLRP3* mutations were initially reported in cryopyrin-associated periodic fever syndrome (CAPS), which includes familial cold autoinflammatory syndrome, Muckle-Wells syndrome and neonatal-onset multisystem inflammatory disease (7). The NLRC4 inflammasome is activated by flagellin and two components of the type III secretion system, the rod and needle proteins, which directly interact with NAIP proteins in a receptor–ligand fashion (9–14). Once the NAIP protein binds its specific ligand, it can bind to NLRC4, leading to NLRC4 oligomerization (13, 14). Hyperactivation of NLRC4 by genetic mutation causes autoinflammatory disorders characterized by CAPS, enterocolitis, or macrophage activation syndrome (15–18), which we hereafter call NLRC4-dysregulated diseases. Patients with mutations in *NLRC4* exhibit increases in IL-18 and IL-1β compared to patients with diseases associated with mutations in *NLRP3*. Indeed, blockade of IL-18 by IL-18BP ameliorates disease severity in patients with *NLRC4* mutations (19, 20).

Here, we assessed the contributions of IL-1β and IL-18 to NLRC4-dysregulated disorders. Mice that harbor a hyperactive *Nlrc4* gene spontaneously develop inflammation in the skin, liver and bone with an increased number of neutrophils in the spleen (15). As reported here, deletion of the *Il1b* gene partially ameliorated bone marrow inflammation and reduced serum IL-18. The deletion of *Il18* completely abolished bone marrow inflammation. In contrast, *Il1b* but not *Il18* deficiency abolished tail bone deformity and reduced bone density. These data demonstrate the distinct roles of IL-1β and IL-18 in NLRC4-dysregulated diseases and reveal that blockade of both IL-18 and IL-1β is needed to completely suppress inflammation.

## Materials and Methods

### Mice

The Nlrc4-H443P-Tg mice, *Il1b*-deficient mice, and *Il18*-deficient mice have been previously reported (15, 21, 22). All mice were maintained under specific pathogen-free conditions in the animal facilities at Tokushima University, Japan. All experiments were performed in accordance with institutional guidelines and the animal care research committee at Tokushima University.

### ELISA

Cytokines in the serum were analyzed using a Mouse G-CSF Quantikine ELISA kit (catalog #MCS00) and mouse IL-18/IL-1F4 ELISA kit (catalog #7625) according to the manufacturer’s instructions (R&D Systems, MN, USA).

### Flow Cytometry

Spleen single-cell suspensions were obtained and treated with RBC lysis buffer. Cells were then incubated with rat anti-mouse CD16/CD32 Ab followed by mAbs specific for extracellular markers. Fluorochrome-conjugated monoclonal antibodies specific for mouse TCRβ (H57-597), CD19 (1D3), CD11b (M1/70), and Gr-1 (RB6-8C5) were purchased from BioLegend (San Diego, CA, USA). Neutrophils were identified as CD11b^+^Gr-1^high^ cells. Data were collected on a FACS Canto II (BD Biosciences) flow cytometer and analyzed using FACS Diva (BD Biosciences) or FlowJo (Tree Star, OR, USA) software.

### Histology

Ear skin samples were collected and fixed in 10% formalin solution. Bone tissues were fixed in 10% formalin and then demineralized in 10% EDTA. The samples were sectioned and stained with H&E and evaluated with respect to cell influx and edema (0, no influx or edema; 1, mild influx and edema; 2, moderate influx and edema; and 3, severe influx and edema) and bone marrow hyperplasia and bone deformity (0, no hyperplasia or deformity; 1, mild hyperplasia and deformity; 2, moderate hyperplasia and deformity; and 3, severe hyperplasia and deformity) by semiquantitative examination.

### Computed Tomography

The left foot and tail were analyzed *in vivo* with microcomputerized tomography at high resolution. The bone density of lower limbs was calculated using Latheta software (Latheta LCT-200, Hitachi Aloka Medical, Tokyo, Japan) 3D morphology reconstruction was performed using 3D Slicer (version 4.10.2). The 3D sections of paw and tail evaluated with respect to bone deformity (0, no deformity, 1, mild; 2, moderate; and 3, severe deformity) by semiquantitative examination.

### Statistical Analysis

For all experiments, the significant differences between groups were calculated using Student’s *t*-test for unpaired data or one-way ANOVA. Differences were considered significant when *p* < 0.05.

## Results

### Both IL-1β and IL-18 Are Required for Inflammation in Nlrc4-H443P-Tg Mice

**Figure 1:**
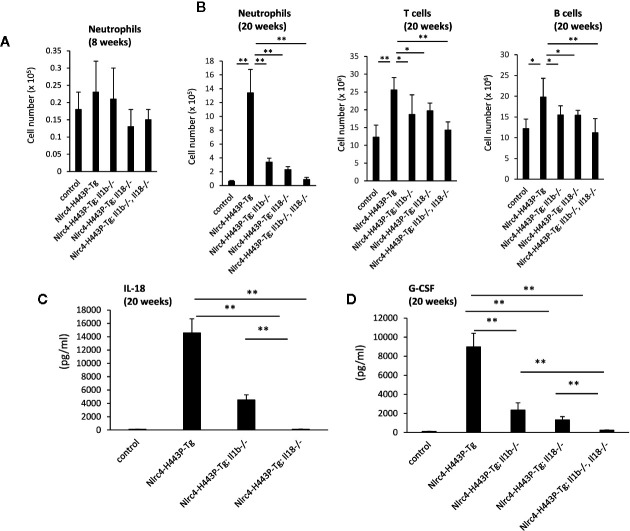
IL-1β– and Il-18–mediated inflammation in Nlrc4-H443P-Tg mice. **(A)** Total neutrophil numbers in the spleens of control, Nlrc4-H443P-Tg, Nlrc4-H443P-Tg/Il1b^−/−^, Nlrc4-H443P-Tg/Il18^−/−^, and Nlrc4-H443P-Tg/Il1b^−/−^Il18^−/−^ mice at the age of 8 and weeks were determined (N = 8 in each group). The neutrophils were defined as CD11b+Gr-1^high^ cells. The data are shown as the mean ± SD. *P < 0.05; **P < 0.01. **(B)** Total neutrophil, T cells or B cells numbers in the spleens of control, Nlrc4-H443P-Tg, Nlrc4-H443P-Tg/Il1b^−/−^, Nlrc4-H443P-Tg/Il18^−/−^, and Nlrc4-H443P-Tg/Il1b^−/−^Il18^−/−^ mice at the age of 20 weeks were determined (N = 8 in each group). The data are shown as the mean ± SD. *P < 0.05; **P < 0.01. Serum **(C)** IL-18 was measured in control, Nlrc4-H443P-Tg, Nlrc4-H443P-Tg/Il1b^−/−^ and Nlrc4-H443P-Tg/Il18^−/−^ mice at the age of 20 weeks (N = 5 in each group), and serum **(D)** G-CSF was measured in control, Nlrc4-H443P-Tg, Nlrc4-H443P-Tg/Il1b^−/−^, Nlrc4-H443P-Tg/Il18^−/−^, and Nlrc4-H443P-Tg/Il1b^−/−^Il18^−/−^ mice at the age of 20 weeks (N = 5 in each group). Data are shown as the mean ± SD. **P < 0.01.

To assess the involvement of IL-1β and IL-18 in Nlrc4-H443P-Tg mice, we crossed Nlrc4-H443P-Tg mice with *Il1b-* or *Il18*-deficient (Nlrc4-H443P-Tg/Il1b^−/−^ or Nlrc4-H443P-Tg/Il18^−/−^, respectively) mice or with mice deficient in both genes (Nlrc4-H443P-Tg/Il1b^−/−^Il18^−/−^). We have previously reported that Nlrc4-H443P-Tg mice have an increased number of CD11b^+^Gr-1^high^ neutrophils in the spleen (15). We first evaluated the number of CD11b^+^Gr-1^high^ neutrophils in the spleens of Nlrc4-H443P-Tg/Il1b^−/−^, Nlrc4-H443P-Tg/Il18^−/−^, and Nlrc4-H443P-Tg/Il1b^−/−^Il18^−/−^ mice at 8 weeks (**Figure 1A**). The number of CD11b^+^Gr-1^high^ neutrophils at 8 weeks was comparable among control, Nlrc4-H443P-Tg/Il1b^−/−^, Nlrc4-H443P-Tg/Il18^−/−^, and Nlrc4-H443P-Tg/Il1b^−/−^Il18^−/−^ mice (**Figure 1A**). An increase in CD11b^+^Gr-1^high^ neutrophils was detected in Nlrc4-H443P-Tg mice at 20 weeks (**Figure 1B**). The number of CD11b^+^Gr-1^high^ neutrophils was reduced in both Nlrc4-H443P-Tg/Il1b^−/−^ and Nlrc4-H443P-Tg/Il18^−/−^ mice, and the number in Nlrc4-H443P-Tg/Il1b^−/−^Il18^−/−^ mice was almost equivalent to that in wild-type mice (**Figure 1B**). The number of T cells and B cells are also increased in Nlrc4-H443P-Tg mice, which was reduced in the absence of IL-1β and IL-18 (**Figure 1B**). In further experiments, to analyze the effect of IL-1β and IL-18, we used mice at the age of 20 weeks.

We next tested the serum levels of cytokines in mice at the age of 20 weeks. IL-18 was increased in the Nlrc4-H443P-Tg mice, and *Il1b* deficiency reduced the level (**Figure 1C**), indicating that the increase in IL-18 is partially dependent on IL-1β. The serum G-CSF level was also increased in the Nlrc4-H443P-Tg mice, and deficiency in either *Il1b* or *Il18* reduced the level, whereas deletion of both led to a level equivalent to that in control mice (**Figure 1D**).

### Blockade of IL-1β or IL-18 Ameliorates Inflammation in Nlrc4-H443P-Tg Mice

**Figure 2:**
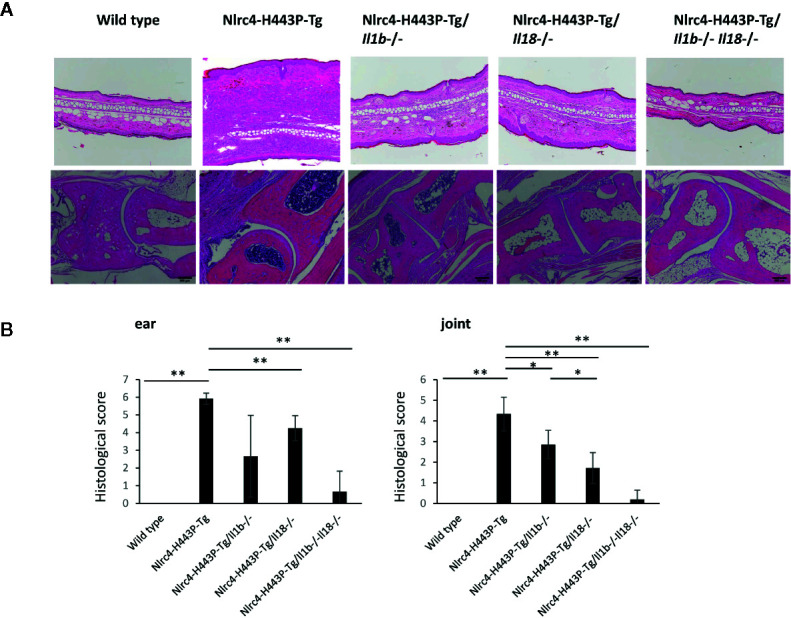
IL-1β– and Il-18–mediated inflammation in the skin and joints of Nlrc4-H443P-Tg mice. **(A)** Histological sections of ear skin and ankle joints in control, Nlrc4-H443P-Tg, Nlrc4-H443P-Tg/Il1b^−/−^, Nlrc4-H443P-Tg/Il18^−/−^, and Nlrc4-H443P-Tg/Il1b^−/−^Il18^−/−^ mice at the age of 20 weeks were stained with hematoxylin and eosin. **(B)** The histological scores were evaluated in the ear skin and ankle joints in in control, Nlrc4-H443P-Tg, Nlrc4-H443P-Tg/Il1b^−/−^, Nlrc4-H443P-Tg/Il18^−/−^, and Nlrc4-H443P-Tg/Il1b^−/−^Il18^−/−^ mice at the age of 20 weeks (N = 8 in each group). Data are shown as the mean ± SD. *P < 0.05, **P < 0.01.

We next analyzed the histology of ear skin, joint and bone marrow samples from control, Nlrc4-H443P-Tg, Nlrc4-H443P-Tg/Il1b^−/−^, Nlrc4-H443P-Tg/Il18^−/−^, and Nlrc4-H443P-Tg/Il1b^−/−^Il18^−/−^ mice (**Figures 2A, B**). The Nlrc4-H443P-Tg mice showed massive infiltration of immune cells in the ear skin, joint and bone marrow samples. The skin in Nlrc4-H443P-Tg mice was thickened because of edema compared with that in wild-type mice. Deletion of either *Il1b* or *Il18* ameliorated cell infiltration in the skin and joints (**Figures 2A, B**). In contrast, deletion of *Il18* almost completely abolished hyperplasia in the bone marrow, while mice deficient in *Il1b* still exhibited moderate hyperplasia in the bone marrow. Deletion of both *Il1b* and *Il18* completely abolished inflammation in any tissues (**Figures 2A, B**). These data indicate that both IL-1β and IL-18 are responsible for inflammation in Nlrc4-H443P-Tg mice and that IL-18 has a greater impact on bone marrow inflammation than IL-1β.

### Blockade of IL-1β Is More Effective Than That of IL-18 in Treating Bone Erosion in Nlrc4-H443P-Tg Mice

**Figure 3:**
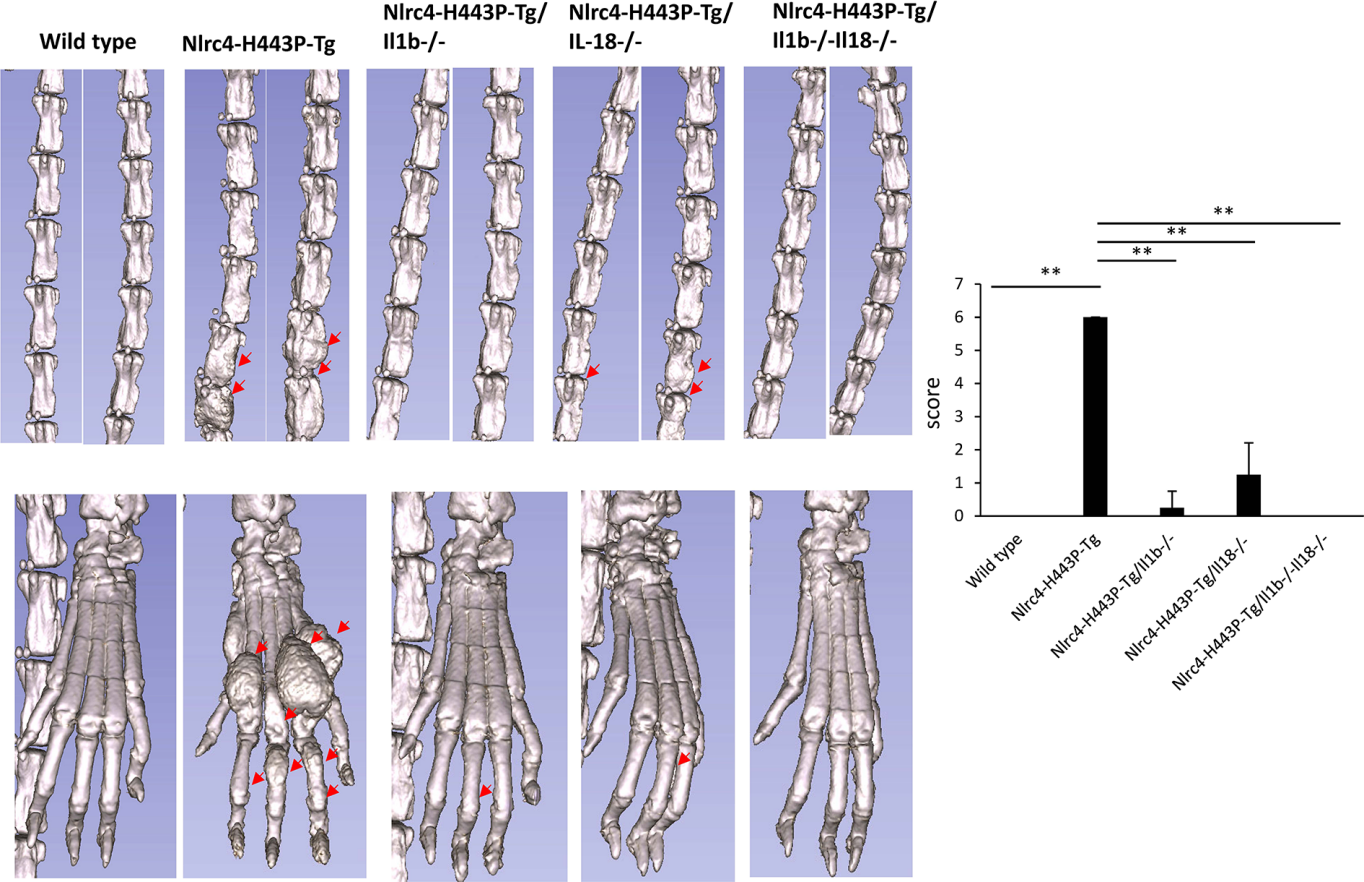
IL-1β– and Il-18–mediated bone deformity in Nlrc4-H443P-Tg mice. The tail and left foot in control, Nlrc4-H443P-Tg, Nlrc4-H443P-Tg/Il1b^−/−^, Nlrc4-H443P-Tg/Il18^−/−^, and Nlrc4-H443P-Tg/Il1b^−/−^Il18^−/−^ mice at the age of 20 weeks were evaluated with μCT. Red arrows, bone deformity regions (N = 5 in each group). The scores were evaluated as shown in the Materials and Methods in control, Nlrc4-H443P-Tg, Nlrc4-H443P-Tg/Il1b^−/−^, Nlrc4-H443P-Tg/Il18^−/−^, and Nlrc4-H443P-Tg/Il1b^−/−^Il18^−/−^ mice at the age of 20 weeks (N = 8 in each group). Data are shown as the mean ± SD. **P < 0.01.

We assessed the shapes of bones in the left foot and tail using μCT analysis to evaluate the effect of IL-1β or IL18 on the bone phenotype of Nlrc4-H443P-Tg mice (**Figure 3**). Bone deformity was also observed in vertebrae (data not shown). Nlrc4-H443P-Tg mice developed severe deformities of the left foot and tail with narrowing of the joint cavity. Deficiency in *Il1b* or *Il18* completely diminished the deformity in the foot bones and joints. In contrast, mice deficient in *Il18* showed mild deformity in the tail bones, while *Il1b*-deficient mice exhibited no tail bone deformity (**Figure 3**).

**Figure 4:**
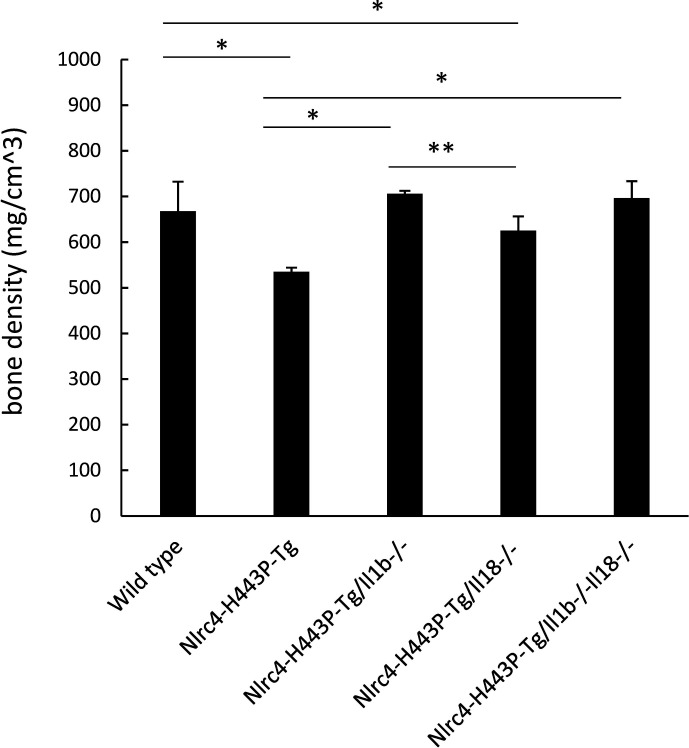
Rescue of decreased bone density by deficiency of Il1b or Il18 in Nlrc4-H443P-Tg mice. Bone density in control, Nlrc4-H443P-Tg, Nlrc4-H443P-Tg/Il1b^−/−^, Nlrc4-H443P-Tg/Il18^−/−^, and Nlrc4-H443P-Tg/Il1b^−/−^Il18^−/−^ mice at the age of 20 weeks was evaluated with μCT (N = 10 in each group). The data are shown as the mean ± SD. *P < 0.05, **P < 0.01.

Bone density was also decreased in Nlrc4-H443P-Tg mice (**Figure 4**). *Il1b* deficiency completely rescued the reduced bone density in Nlrc4-H443P-Tg mice, while *Il18* deficiency partially reversed this reduction (**Figure 4**). These data suggest that IL-1β has more important roles in bone phenotypes than IL-18 in NLRC4-dysregulated diseases.

## Discussion

Heterozygous mutations in *NLRC4* cause several types of autoinflammatory disorders. For instance, *NLRC4* mutations cause CAPS-like syndrome, including cold-induced autoinflammatory syndrome and NOMID (15, 18). The clinical phenotypes of CAPS-like syndrome caused by *NLRC4* mutations are indistinguishable from those caused by *NLRP3* mutations. *NLRC4* mutations also cause infantile enteritis and macrophage activation syndrome (16, 17). One of the characteristics of NLRC4-dysregulated diseases is high serum IL-18, and IL-18 blockade is effective in treating macrophage activation syndrome caused by *NLRC4* mutation (19, 20). However, the roles of IL-18 and IL-1β in NLRC4-dysregulated diseases are not fully understood, and thus, we tested the roles of each cytokine by using mice in which a mutant *Nlrc4* is expressed under the MHC class II promoter in combination with *Il1b* and *Il18* deficiency. Our data demonstrated the distinct roles of IL-1β and IL-18 in inflammation in the joints and skin, suggesting that the efficacy of blockers of each cytokine depends on the regions of inflammation.

Blockade of IL-1β and Il-18 is effective for ameliorating inflammation in Nlrc4-H443P-Tg mice, although the contribution of each cytokine to inflammation is distinct among tissues. Induced deficiency of *Il18* is more effective than that of *Il1b* in suppressing bone marrow hyperplasia, and *Il1b* deficiency better inhibited bone deformity and decreases in bone density than *Il18* deficiency. One possibility to explain the distinct effects of these cytokines in tissues would be differences in the cell types that respond to IL-1β or IL-18. In any case, our data suggest that the blockade of both IL-1β and IL-18 is required to completely suppress inflammation in NLRC4-dysregulated diseases. In addition, Nlrc4-H443P-Tg mice with deletion of both *Il1b* and *Il18* do not show any inflammation or any increase in neutrophils, suggesting a small contribution of pyroptosis-mediated cell death and cell death-associated damage-associated molecular patterns to inflammation in NLRC4-dysregulated diseases.

NOMID patients exhibit bone deformity accompanied by joint inflammation (23, 24). Nlrc4-H443P-Tg mice also show severe bone deformity at the age of 20 weeks. Bone deformity is caused by IL-1β and IL-18, but blockade of IL-18 is not enough to suppress the deformity. On the other hand, recent studies have indicated that the blockade of IL-18 is effective in treating macrophage activation syndrome caused by *NLRC4* mutation that is refractory to blockade of IL-1β. Our data suggest that bone phenotypes in NLRC4-associated NOMID might be treated with a combination of blockers of IL-1β and IL-18. Regarding the contribution of IL-1β and IL-18 to bone deformity, a previous study demonstrated that IL-18 upregulates membrane-bound receptor activator of nuclear factor kappa B ligand (RANKL) expression and soluble RANKL production, thus increasing the ratio of RANKL/osteoprotegerin, suggesting an effect of IL-18 on the induction of osteoclast formation and bone resorption (25). IL-1β is involved in osteoclast differentiation in part through the induction of TRAF-6 downstream of the IL-1β pathway (26). Further studies are required to clarify not only the molecular mechanism of IL-1β-mediated bone deformity but also the roles of IL-18 in this context.

Several gene-modified mice were reported to investigate the molecular mechanisms of autoinflammatory disorders including CAPS. Pyrin-knock-in mice harboring mutant human B30.2 domains exhibited spontaneous bone marrow-dependent inflammation similar to that seen in human familial Mediterranean fever (27). This inflammation was completely abrogated in the absence of the IL-1 receptor or the adaptor molecule ASC. Nlrp3-knock-in mice demonstrated early mortality mediated by myeloid cells, which was only partially dependent on IL-1β (28). Deletion of *Il18r* in Nlrp3-knock-in mice resulted in partial phenotypic rescue in skin and visceral disease and reduced serum cytokines (29). Another strain of Nlrp3-knock-in mice exhibited skin inflammation with neutrophil infiltration and a Th17 cytokine-dominant response, which was suppressed in the absence of the IL-1 receptor (30). The comparison of our Nlrc4-H443P-Tg mice with the pyrin- or Nlrp3-knock-in mice side by side would be interesting to understand the effect of each cytokine on organ pathology induced by *NLRC4* mutations.

In summary, our data demonstrate the crucial contributions of IL-1β and IL-18 to NLRC4-dysregulated diseases but reveal that the two cytokines have distinct roles depending on the tissue. These data suggest that blockers of IL-1β and IL-18 should be utilized depending on the site of tissue inflammation in NLRC4-dysregulated diseases.

## Data Availability Statement

The raw data supporting the conclusions of this article will be made available by the authors, without undue reservation.

## Ethics Statement

The animal study was reviewed and approved by animal care research committee at Tokushima University.

## Author Contributions

YS and KY designed the studies. YS, KO, SI T, and HA analyzed the data. YS and KY wrote the paper. KY supervised all studies. All authors contributed to the article and approved the submitted version.

## Funding

The research is supported by The Research Cluster Program on Immunological Diseases, Tokushima University

## Conflict of Interest

The authors declare that the research was conducted in the absence of any commercial or financial relationships that could be construed as a potential conflict of interest.

## Supplementary Material

Supplementary Figure 1
*Gating strategy for detecting neutrophils*. Total spleen cells were stained with 7AAD, anti-CD11b and Gr-1 antibodies. In the singlet and 7AAD negative populations, CD11b+Gr-1^high^ cells were defined as neutrophils.
