# Supplementary figures and images for: Distinct Roles of IL-1β and IL-18 in NLRC4-Induced Autoinflammation
Source: Front Immunol. 2020 Oct 14;11:591713. doi: 10.3389/fimmu.2020.591713 (PMC7592392; doi:10.3389/fimmu.2020.591713)

Supplementary Figure 1

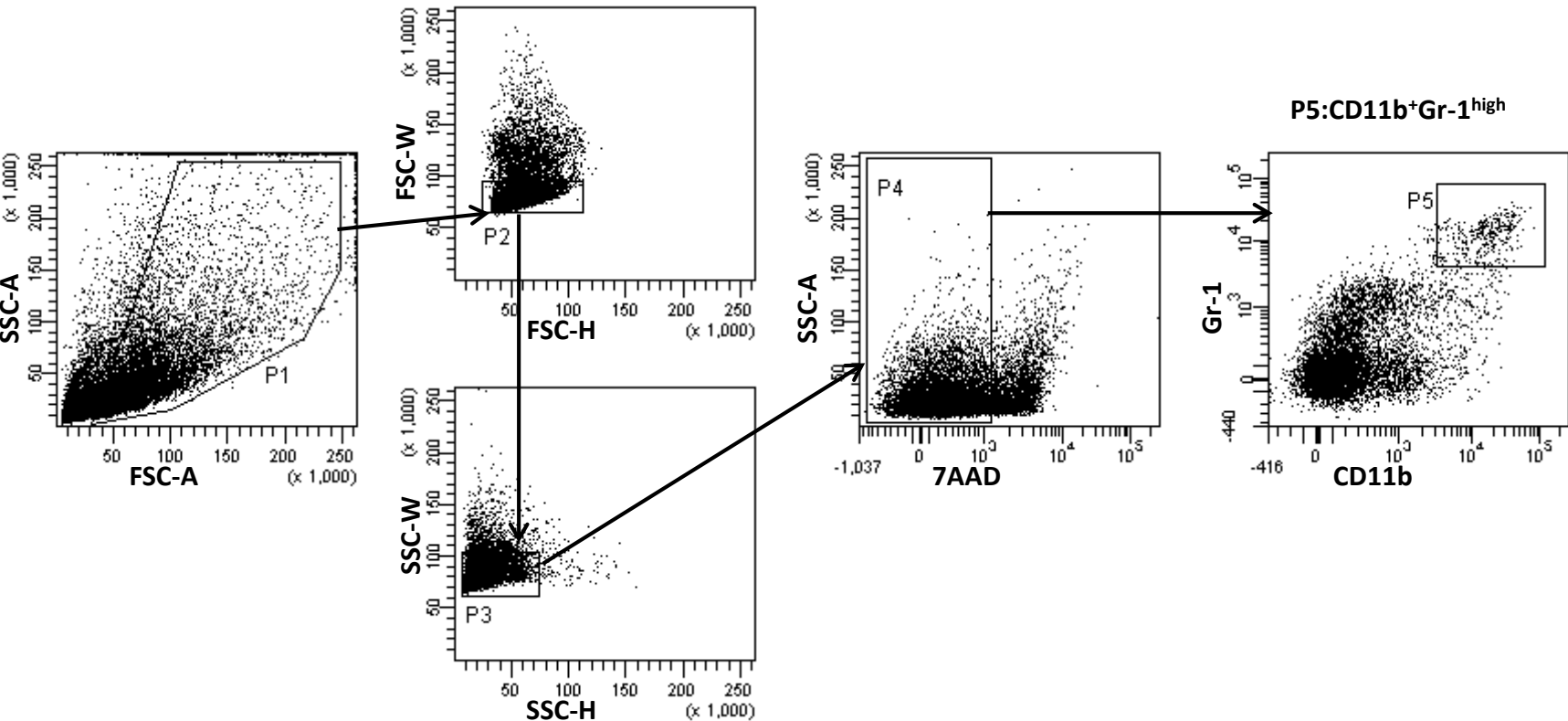

Supplement: Supplementary Figure 1 — Gating strategy for detecting neutrophils. Total spleen cells were stained with 7AAD, anti-CD11b and Gr-1 antibodies. In the singlet and 7AAD negative populations, CD11b+Gr-1high cells were defined as neutrophils. [file DataSheet_1.pdf]
